# Chemotherapy-related striate melanonychia: a case report

**DOI:** 10.1186/s13256-020-02612-5

**Published:** 2021-01-31

**Authors:** Fazleenah Hussain, Dushyanth Gnanappiragasam, Freida Shaffrali

**Affiliations:** 1 Department of Dermatology, University Hospital Monklands, Airdrie, ML6 0JS UK; 2 Department of Dermatology, University Hospital of Leicester NHS Trust, Leicester, LE1 5WW UK

**Keywords:** Striate melanonychia, Chemotherapy, Nail pigmentation, Case report

## Abstract

**Background:**

Chemotherapy medications are reported to cause discoloration of the nails known as melanonychia. Depending on the nail structure affected and the severity of the insult, the clinical features can be variable. There are a great deal of unreported cases of pigmentary nail changes associated with chemotherapy treatment. By sharing our knowledge, we hope to raise the awareness of these nail changes amongst clinicians. Early recognition is crucial to allay anxiety among patients and avoid any unnecessary investigations.

**Case presentation:**

We present a case of 36-year-old woman of south Asian origin, who developed dark pigmentation in the left thumb nail during neoadjuvant chemotherapy with 5-fluorouracil, epirubicin, cyclophosphamide, and docetaxel (FEC-D) for triple negative breast cancer. Upon examination, the left thumb nail pigmentation was strikingly linear, uniform, and well demarcated extending from proximal nail fold to free margin. Despite the reassuring clinical features, the patient was understandably anxious that this could be a presentation of acral melanoma and was referred to the plastic surgeons for a nail matrix biopsy. Biopsy reassuringly was reported as melanosis and a diagnosis of striate melanonychia was made. The patient was discharged after 2-year follow-up.

**Conclusion:**

Chemotherapy medications have improved survival rates and patient outcomes. It is important for clinicians to be aware of the association of melanonychia with certain chemotherapy medications to reduce anxiety and allow successful management of these patients without delay. Striate melanonychia in this patient was felt most likely due to the synergistic effect of chemotherapy drugs compounded with racial predisposition. Chemotherapy agents most likely to have contributed include cyclophosphamide, docetaxel, and 5-fluorouracil.

## Background

Melanonychia is defined as brown or black discoloration of the finger or toe nails. There are a multitude of causes for melanonychia. Depending on the nail structure affected and the severity of the insult, the clinical features can be variable. Nail changes can involve single, multiple, or all of the nails. A thorough history, direct clinical and dermatoscopy examination usually aid in diagnosis; however, histology examination remains the gold standard investigation. Chemotherapy medications have been associated with a variety of nail changes and infrequently reported to cause melanonychia. The diagnosis and management of chemotherapy medications related to melanonychia can often present a unique challenge to clinicians. Benign causes of melanonychia most often benefit from watch and wait approach. Any suspicious malignant lesions should be excised and melanonychia due to infectious causes managed with antimicrobials. We present a case of 36-year-old female who developed dark pigmentation in the left thumb nail during neoadjuvant chemotherapy with 5-fluorouracil, epirubicin, cyclophosphamide, and docetaxel (FEC-D) for triple negative breast cancer. Our patient had a nail biopsy which showed melanosis with no evidence of atypia. She was discharged after a period of follow-up.

## Case presentation

We present a case of a 36-year-old Asian female who initially noticed a right breast lump. She was reviewed by the breast surgeons and biopsy confirmed triple negative (estrogen receptor, progesterone receptor, and human epidermal growth factor receptor 2) breast cancer. Ultrasound-guided biopsy of the right axillary node was negative. Neoadjuvant FEC-D chemotherapy was commenced together with monthly goserelin as ovarian protection with eight cycles given in total. She also received granulocyte colony-stimulating factor (G-CSF) to prevent neutropenia.

She tolerated the FEC-D chemotherapy well apart from an episode of grade 1 thrush and migraine. This was treated with nystatin, fluconazole, and co-codamol. She also attended the accident and emergency department for urinary tract infection and was prescribed trimethoprim. She was not on other medications at that time. She received adjuvant two-field radiotherapy and a boost to increase the amount of radiation delivered to the area at highest risk of breast cancer recurrence. She underwent wide local excision and sentinel lymph node biopsy 5 months after initiation of chemotherapy.

The patient noticed dark pigmentation of her left thumb nail a few days after the second cycle of chemotherapy. Over the next few months, the linear pigmentation on her thumb nail became darker. She denied pain, bleeding, or preceding history of trauma. There was no toenail involvement. She did not have any previous skin disease including psoriasis or lichen planus.

**Fig. 1:**
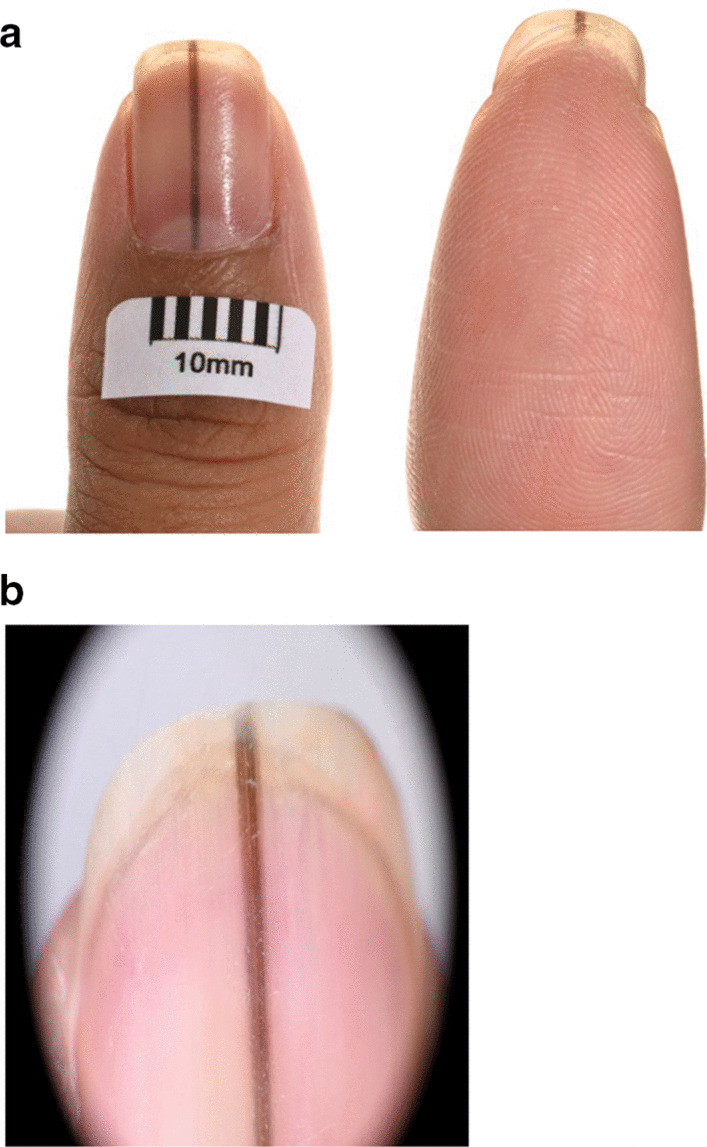
**a** Anterior and posterior views of the left thumb. **b** Dermatoscopic view of the left thumb

On presentation to the dermatology department, the left thumb nail pigmentation was linear, uniform, and well demarcated extending from proximal nail fold to free margin (Fig. 1a, b). Hutchinson’s sign was negative. No other nails were involved, and no rash noted elsewhere. There was no pigmentation of the oral mucosa. Patient was otherwise clinically well. Blood tests including full blood count, renal profile, liver function test, HbA1c, thyroid function test, erythrocyte sedimentation rate, lipid profile, coagulation profile, B12, folate, iron studies, phosphate, and magnesium were all in the normal range.

**Fig. 2:**
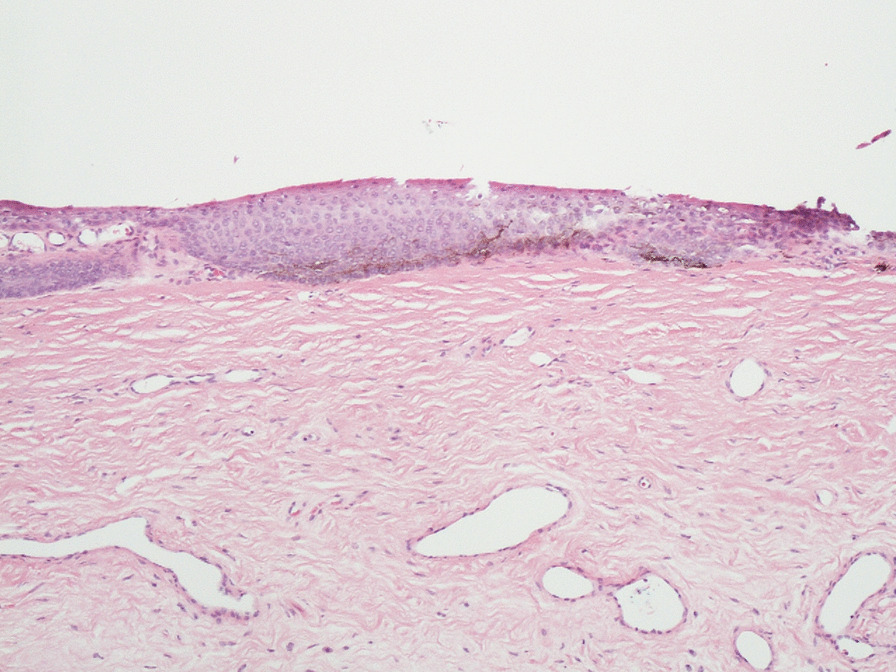
Hematoxylin and eosin stain, ×100

**Fig. 3:**
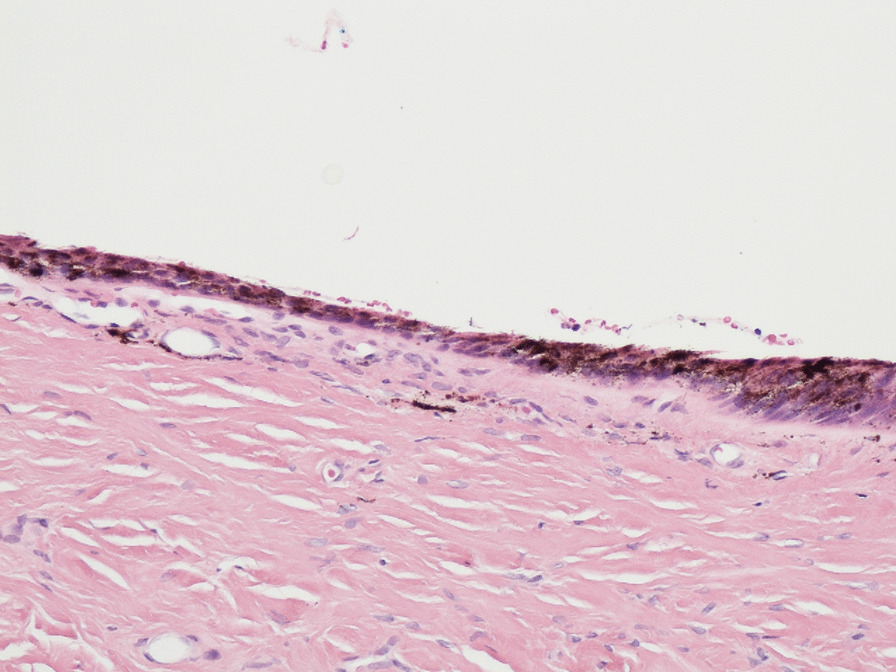
Hematoxylin and eosin stain, ×200

**Fig. 4:**
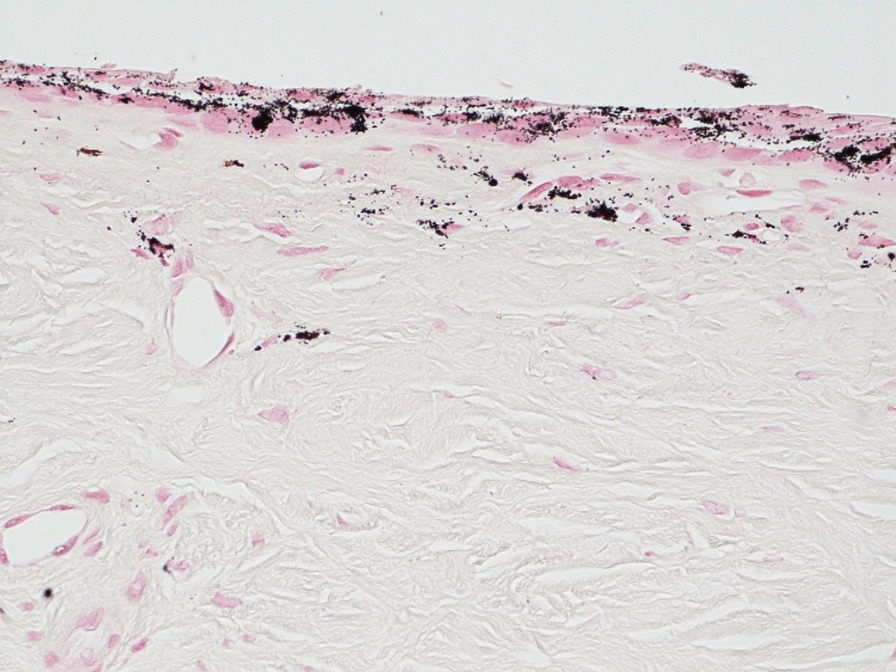
Masson Fontana stain, ×200

At 12-month follow-up after initial review, the appearance was stable and was suggestive of striate melanonychia. At 24 months follow-up, the changes remained stable. Despite the reassuring clinical features, the patient was understandably anxious whether this might be a presentation of acral melanoma given her history and was referred to the plastic surgeons for a nail matrix biopsy. Biopsy reassuringly was reported as melanosis (Figs. 2, 3, 4). Figures 2 and 3 show evidence of central pigmentation which was confirmed as melanin with positive staining with Masson Fontana (Fig. 4). There is no evidence of significant atypia noted.

Striate melanonychia in this lady was felt most likely due to the synergistic effect of chemotherapy drugs compounded with racial predisposition. Chemotherapy agents most likely to have contributed include cyclophosphamide, docetaxel, epirubicin and 5-fluorouracil (Tables 1 and 2) [1, 2].

**Table 1:** Drugs associated with longitudinal melanonychia [1]

Chemotherapeutical	Bleomycin sulfate, busulfan, cyclophosphamide, dacarbazine, daunorubicin hydrochloride, doxorubicin, etoposide, 5-fluorouracil, hydroxyurea, imatinib, melphalan hydrochloride, methotrexate, nitrogen mustard, nitrosourea, tegafur
Antibacterial	Sulfonamide, minocycline, roxithromycin, clofazimine
Antifungal	Ketoconazole, fluconazole, voriconazole
Antimalarial	Chloroquine, mepacrine, amodiaquine
Others	ACTH, amorolfine, arsenic, chloroquine, clomipramine, cyclones, fluorides, gold salts, ibuprofen, lamivudine, mercury, MSH, PCB, phenytoin, phenothiazine, psoralen, steroids, thallium, timolol, zidovudine

*ACTH* adrenocorticotropic hormone,* MSH* melanocyte‐stimulating hormone,* PCB* polychlorinated biphenyl

**Table 2:** List of drugs relevant to our case and their association with melanonychia (*Litt’s Drug Eruption Manual*, 22nd edition) [2]

Cyclophosphamide	Melanonychia, nail pigmentation
Docetaxel	Melanonychia, nail pigmentation
Epirubicin	Nail pigmentation
5-Fluorouracil	Nail pigmentation
Goserelin	No nail pigmentary changes reported
Metoclopramide	No nail pigmentary changes reported
Ondansetron	No nail pigmentary changes reported
Dexamethasone	No nail pigmentary changes reported

## Discussion

Nail changes often provide important diagnostic clues and can be seen in inflammatory conditions, systemic diseases, diseases of specific organ systems, or associated with syndromes and genodermatoses. For example, koilonychia is seen in iron deficiency anemia, pitting of nails seen in psoriasis, and V-shaped notch can be seen in Darier’s disease [3].

Melanonychia is characterized by brown to black discoloration of the nail plate [4]. Melanonychia can be associated with genetic disorders, systemic diseases, nutritional deficiencies, connective tissue disorders, inflammatory skin disease, medications, nail injury, trauma and infection . Melanonychia can be a result of melanocytic hyperplasia or melanocytic activation (Table 3). Striata melanonychia and longitudinal melanonychia are synonymous terms used in the literature and in most case reports. Longitudinal melanonychia is the most common form of the presentation and transverse melanonychia and diffuse melanonychia are also infrequently encountered [5].

**Table 3:** Conditions associated with melanonychia due to melanocytic activation and melanocytic hyperplasia in the nail matrix [1]

Melanocytic activation in the nail matrix	
Physiologic causes	Racial Pregnancy
Local and regional causes	Repeated local trauma from poor footwear or overriding toes Onychotillomania Nail biting Occupational trauma Carpal tunnel syndrome
Dermatologic causes	Onychomycosis Chronic paronychia Psoriasis Lichen planus Amyloidosis Chronic radiation dermatitis Systemic lupus erythematosus Localized scleroderma Onychomatricoma Bowen’s disease Myxoid pseudocyst Basal cell carcinoma Subungual fibrous histiocytoma Verruca vulgaris Subungual linear keratosis
Systemic causes	Endocrine (Addison’s, Cushing’s syndrome) Nelson’s syndrome, hyperthyroidism, acromegaly Alcaptonuria Nutritional disorders Hemosiderosis Hyperbilirubinemia Porphyria Graft versus host disease AIDS
Iatrogenic causes	Phototherapy X-ray exposure Electron beam therapy Drug intake
Syndromes	Laugier-Hunziker syndrome Peutz-Jeghers syndrome Touraine syndrome

Melanocytic hyperplasia in the nail matrix	
Benign	Congenital nevi Acquired nevi Nail lentigo
Neoplasm	Subungual melanoma in situ Subungual melanoma Subungual pigmented Bowen disease Subungual pigmented squamous cell carcinoma

Melanocytic activation is an increase in the activity of melanocytes resulting in increased melanin synthesis in the nail matrix and deposition in the nail plate. A wide range of factors can induce melanocytic activation. Drug-induced melanonychia is hypothesized to be due to melanocytic activation combined with increased melanin synthesis and deposition [6]. Chemotherapy medications can be associated with a variety of nail changes including melanonychia, onychomadesis, onycholysis, leukonychia, Beau’s line, nail plate abnormalities (brittle or thin nail), alteration of nail growth rate and subungual hemorrhage.

Nail matrix contains actively dividing cells and can be affected by the antimitotic property of the chemotherapy medications as a result [7]. In a study consisting of 150 patients undergoing chemotherapy, pigmentary nail changes were the common nail change reported [5]. Longitudinal melanonychia was seen in 67.7% of patients on platinum-based chemotherapeutic agents and 16.1% of patients on the cyclophosphamide, doxorubicin, vincristine, and prednisolone (CHOP) regime in this study. Transverse and diffuse melanonychia patterns have also been described in patients undergoing chemotherapy treatment [8].

Melanocytic hyperplasia refers to increase in the proliferation of melanocytes in the nail matrix and nail plate. It can be induced by benign or malignant causes. Often in patients presenting with any form of melanonychia, subungual melanoma needs to be considered and ruled out. Subungual melanoma usually carries a poor prognosis. Late presentation could be a potential contributing factor for the delay in diagnosis. Variation in width, longitudinal band with either triangular or pyramidal shape, pigment heterogeneity, Hutchinson’s sign, symptoms such as pain, bleeding, and persistent secondary infection can be features associated with suspected subungual melanoma [9]

As listed in Table 3, melanonychia can also be seen commonly in some racial types. Darker-skinned people such as black, Asian, Hispanic, and Middle Eastern individuals frequently have benign longitudinal melanonychia [2, 10]. It is plausible that in dark-skinned patients, such as in our case, they may already had subtle early melanonychia changes to some degree even before commencing on new therapeutics which may not have been noticed before. Starting on new therapeutics such as chemotherapy medications perhaps contributes towards the accentuation of the melanonychia and it becoming visibly noticeable.

There is no effective treatment to reverse melanonychia due to the transient impairment and arrest of nail matrix mitotic activity. Some nail abnormalities often would improve over time with nail growth. It is imperative to educate patients regarding the potential melanonychia changes and providing practical strategies where applicable.

## Conclusion

Chemotherapy medications have improved survival rates and patient outcomes. It is important for clinicians to be aware of the association of melanonychia with certain chemotherapy medications to reduce anxiety and allow successful management of these patients without delay. If in doubt, early dermatological review should be considered.

## Data Availability

Data sharing not applicable to this article as no datasets were generated or analyzed during the current study.
